# Association of methylation risk score with incident type 2 diabetes mellitus: A nested case–control study

**DOI:** 10.1111/1753-0407.13512

**Published:** 2023-12-07

**Authors:** Weifeng Huo, Huifang Hu, Tianze Li, Lijun Yuan, Jinli Zhang, Yifei Feng, Yuying Wu, Xueru Fu, Yamin Ke, Mengmeng Wang, Wenkai Zhang, Longkang Wang, Yaobing Chen, Yajuan Gao, Xi Li, Jiong Liu, Zelin Huang, Fulan Hu, Ming Zhang, Liang Sun, Dongsheng Hu, Yang Zhao

**Affiliations:** ^1^ Department of Epidemiology and Biostatistics College of Public Health, Zhengzhou University Zhengzhou China; ^2^ Department of Preventive Medicine School of Public Health, Shenzhen University Medical School Shenzhen China; ^3^ Department of Biostatistics and Epidemiology School of Public Health, Shenzhen University Medical School Shenzhen China; ^4^ Department of Social Medicine and Health Service Management College of Public Health, Zhengzhou University Zhengzhou China

**Keywords:** DNA methylation, interaction analysis, methylation risk score, nested case–control study, type 2 diabetes mellitus

## Abstract

**Aims:**

To investigate the association of methylation risk score (MRS) and its interactions with environmental factors with type 2 diabetes mellitus (T2DM) risk.

**Methods:**

We conducted a nested case–control study with 241 onset cases and 241 matched controls. Conditional logistic regression models were employed to identify risk CpG sites. Simple and weighted MRSs were constructed based on the methylation levels of ATP‐binding cassette G1 gene, fat mass and obesity associated gene, potassium voltage‐gated channel member 1 gene, and thioredoxin‐interacting protein gene previously associated with T2DM to estimate the association of MRS with T2DM risk. Stratified analyses were used to investigate interactions between MRS and environmental factors.

**Results:**

A total of 10 CpG loci were identified from the aforementioned genes to calculate MRS. After controlling for potential confounding factors, taking tertile 1 as reference, the odds ratios (ORs) and 95% confidence intervals (CIs) for T2DM of tertile 3 was 2.39 (1.36–4.20) for simple MRS and 2.59 (1.45–4.63) for weighted MRS. With per SD score increment in MRS, the OR (95% CI) was 1.66 (1.29–2.14) and 1.60 (1.24–2.08) for simple and weighted MRSs, respectively. J‐curved associations were observed between both simple and weighted MRSs and T2DM risks. Additionally, multiplication interactions for smoking and hypertension with simple MRS on the risk of T2DM were found, similarly for smoking and obesity with weighted MRS on the risk of T2DM (all *P*_interaction_ < .05).

**Conclusion:**

Elevated simple and weighted MRSs were associated with increased risk of T2DM. Environmental risk factors may influence the association between MRS and T2DM.

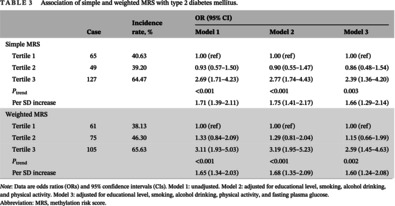

## Graphical abstract

**Highlights**

- This is the first prospective nested case–control study based on the Chinese population to explore the relationship of methylation risk score (MRS) with type 2 diabetes mellitus (T2DM) risk.
- We calculated simple and weighted MRSs based on the methylation levels of 10 risk CpG sites of ATP‐binding cassette G1 gene, fat mass and obesity associated gene, potassium voltage‐gated channel member 1 gene, and thioredoxin‐interacting protein gene before T2DM occurred.
- We found a significant positive association between MRS and the risk of T2DM incidence.

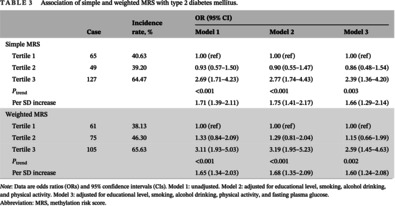

## 1 INTRODUCTION

Diabetes mellitus has emerged as a global health concern, with an estimated 10.5% of adults living with diabetes mellitus globally in 2021. The prevalence of diabetes mellitus is predicted to reach 12.2% in 2045.^1^ In China, there were 140.9 million adults suffering from diabetes mellitus in 2021, which made China the epicenter of the global diabetes mellitus epidemic. Among them, type 2 diabetes mellitus (T2DM) is the most predominant type of diabetes mellitus, accounting for more than 90%.^2^, ^3^ T2DM results in complications, both microvascular and macrovascular, which causes profound damage to the health of patients and puts a huge strain on the health care system.^4^ Measures that contribute to T2DM prevention are therefore urgently needed.

T2DM is a complex disease because the pathogenesis is associated with environmental and genetic factors.^4^ These environmental risk factors include obesity,^5^ smoking,^6^ and physical inactivity,^7^ and mounting evidence suggests that epigenetics contributes significantly to T2DM onset.^8^, ^9^, ^10^, ^11^ Epigenetics determines how the organism responds to risk factors, which may mean that susceptibility to T2DM differs between individuals. Epigenetics includes DNA methylation, histone modifications, and noncoding RNAs, of which DNA methylation has been the most widely studied and best characterized.^12^, ^13^, ^14^ Studies mainly based on European populations have found that the methylation of ATP‐binding cassette G1 (ABCG1) gene, fat mass and obesity associated (FTO) gene, potassium voltage‐gated channel member 1 (KCNQ1) gene, and thioredoxin‐interacting protein (TXNIP) gene were associated with T2DM.^15^, ^16^, ^17^ These associations were confirmed in our previous work.^18^, ^19^, ^20^, ^21^ Although these studies investigated only the association between each gene and T2DM. Most recently, the DNA methylation risk score (MRS), a method of integrating information across genes, has attracted the interest of researchers.^22^ Epigenome‐wide association studies (EWAS) among Europeans and Indian Asians have found that higher MRS was associated with an increase in T2DM incidence.^15^, ^23^ Studies investigating the association between MRS and T2DM in the Chinese population, however, are currently insufficient.

In present study, we constructed MRS based on ABCG1, FTO, KCNQ1, and TXNIP. We explored the association between MRS and the risk of T2DM in a nested case–control study with a Chinese rural population, aiming to provide epidemiological evidence for early identification of individuals at high risk of T2DM.

## 2 METHOD

### 2.1 Study population

T2DM cases and controls were selected for the study from The Rural Chinese Cohort Study (RCCS).^24^ The RCCS enrolled 20 194 adult (≥18 years) participants during 2007 and 2008, acquiring baseline data on individuals through designed questionnaire interviews and anthropometric and laboratory measurements. Details of the RCCS have been described elsewhere.^24^ All individuals in our study agreed to participate voluntarily and provided signed consent. The study protocol was conducted with the approval of the College of Public Health of Zhengzhou University.

During 2013 and 2014 the first follow‐up was conducted, with data on 17 265 participants collected. Individuals were excluded if, at baseline, they had suffered from type 1 diabetes mellitus (*n* = 13), T2DM (*n* = 1499), cancer (*n* = 28), stroke (*n* = 372), myocardial infarction (*n* = 183), chronic obstructive pulmonary disease (*n* = 353), or chronic kidney disease (*n* = 294). Eventually, for this current study, our sample included 14 523 eligible participants. At follow‐up, 707 individuals had developed T2DM. Among them, 293 participants were chosen as the cases through simple random sampling. Then 293 nondiabetic participants were matched (1:1) to cases by age, gender, ethnicity, marital status, and village of residence. All matching factors were exactly matched except for age (±5 years). Finally, DNA methylation level detection was completed in a sample of 572 (97.6%). In addition, we excluded 45 pairs due to their lacking data on methylation levels of ABCG1, FTO, KCNQ1, or TXNIP, eventually employing 241 pairs to estimate the effect of MRS on the risk of incident T2DM.

### 2.2 Data collection

Demographic data (sex, age, marital status, race, village of residence, and educational level), lifestyle data (smoking, alcohol drinking, and physical activity), and personal and family medical histories of disease at baseline and follow‐up were obtained by questionnaire. Participants having smoked ≥100 cigarettes to date were defined as smokers. Participants having consumed alcohol ≥12 times in the past year were defined as alcohol drinkers.^24^ Data on physical activity from the previous year were collected, then participants divided into three levels of physical activity: low, moderate, and high, in accordance with the international physical activity questionnaire.^25^ Participants with first‐degree relatives suffering T2DM were defined as having a family history of T2DM.

Anthropometric data were collected by skilled investigators. Subjects were required to wear light clothes, no shoes, and no heavy items for measurement of weight, height, and waist circumference (WC). Body mass index (BMI) was then calculated according to weight (kg)/height (m^2^).^26^ Individuals with BMI ≥28 kg/m^2^ were characterized as obese.^27^ After resting for at least 5 min, blood pressure (BP) was measured using an electronic sphygmomanometer on the bare right arm three times, with a half minute interval between each measurement. Participants whose mean systolic blood pressure (SBP) ≥ 140 mm Hg and/or mean diastolic blood pressure (DBP) ≥ 90 mm Hg and/or were currently using antihypertensive medication were recognized as hypertense.^28^

After fasting for over 8 h, participants gave overnight blood samples. We used an automatic biochemical analyzer (Hitachi 7060, Tokyo) to measure total cholesterol (TC), triglycerides (TG), high‐density lipoprotein cholesterol (HDL‐C), and fasting plasma glucose (FPG) levels. Low‐density lipoprotein cholesterol (LDL‐C) was computed according to the Friedewald formula.^29^ Individuals with TC level ≥6.62 mmol/L, TG level ≥2.26 mmol/L, LDL‐C level ≥4.14 mmol/L, or HDL‐C level <1.04 mmol/L were identified as having dyslipidemia, employing the criteria in the Guidelines on Prevention and Treatment of Dyslipidemia for Chinese adults.^30^

### 2.3 Definition of T2DM

Individuals were defined as having T2DM if they meet one of the following criteria: (1) having a measurement of FPG ≥7.0 mmol/L, (2) having current use of insulin or oral hypoglycemic agents, or (3) having a self‐reported history of T2DM.^31^

### 2.4 Quantitative DNA methylation measurement

In our earlier studies, the methylation of ABCG1,^18^ FTO,^19^ KCNQ1,^20^ and TXNIP^21^ had been found to be associated with T2DM onset risk, so we constructed MRS based on the aforementioned genes.

DNA extraction was from baseline fasting external blood white blood cells using an automated nucleic acid extraction system (BioTekd Corp., Beijing). We designed the primers for the aforementioned genes using Sequenom EpiDesigner software. For our analysis, the chosen region of ABCG1 sequence employed was chr21: 43656137–43 657 036, which included 15 CpG loci and covered cg06500161; the chosen region of FTO was Chr16: 53703509–53 703 936, which included 19 CpG loci; that of KCNQ1 was chr11:2445193–2 445 759, which included 39 CpG loci and covered cg12578166, cg05418487, cg13071812, cg08303146, and cg15974272; and that of TXNIP was chr1:145441299–145 441 621, which included 5 CpG loci and covered cg19693031.

We treated genomic DNA with bisulfite, then amplified target sequences by polymerase chain reaction (PCR). PCR products were further treated with shrimp alkaline phosphate to remove 5′‐and 3′‐phosphate groups, followed by transcribing into RNA in vitro. Finally, each RNA fragment was cleaved into fractions with CpG loci, using the ability of RNase A to specially identify the U 3′ end of RNA. We used MALDI‐TOF mass spectrometry based on the MassARRAY System (Bio Miao Biological Technology, Beijing) to analyze the mass spectra of products, and MassARRAY EpiTYPER analysis (Agena Bioscience, San Diego, CA) to measure the DNA methylation levels.

### 2.5 CpG sites selection and MRS calculation

To construct MRS, the methylation level of each CpG site was divided into three categories according to the tertiles of score: tertile 1, tertile 2, tertile 3, scoring 0, 1, 2, respectively. Considering that the hypomethylation of TXNIP increases T2DM risk,^21^ TXNIP was scored using 1 minus methylation level of CpG sites of TXNIP. We used a conditional logistic regression model to compute the *p*‐value for every CpG site of participants in tertile 3, taking tertile 1 as reference, and we selected CpG sites that achieved statistical significance (*p* < .05) as risk CpG sites.

Simple MRS for participants was calculated by directly summing the scores of the risk CpG sites (0, 1, or 2) at each gene. To calculate the weighted MRS, odds ratios (ORs) from the risk CpG sites were employed as internal weights to multiply the CpG methylation levels, with the products summed to be the weighted MRS for each participant.^22^

### 2.6 Statistical analysis

Baseline data on 241 pairs were summarized and compared between groups. For continuous variables, data were presented with mean and SD according to normal distribution, median, and interquartile range due to skewed distribution and were analyzed by paired *t* test, or Wilcoxon signed‐rank test, respectively. Categorical variables were reported in number (percentages) and compared using McNemar's test.

Participants were divided into three groups based on tertiles of MRSs. And a conditional logistic regression was applied to calculate the ORs and 95% confidence intervals (95% CIs) across simple MRS and weighted MRS categories with tertile 1 as reference. Three models were performed: model 1 was not adjusted; model 2 was adjusted for educational level, smoking, alcohol drinking, and physical activity; and model 3 was further adjusted for FPG. The dose–response relationships of simple MRS and weighted MRS with T2DM were modeled by restricted cubic splines using three knots: 25th, 50th, and 75th centiles of MRS.

In addition, stratified analyses of simple MRS and weighted MRS were also performed to estimate the potential modification effects on T2DM risk of the following factors: gender (female or male), smoking (ever/current or never), physical activity (low or moderate/high), obesity (yes or no), hypertension (yes or no), and dyslipidemia (yes or no). A receiver operating characteristic (ROC) curve analysis was used to assess the predictive effect of MRSs for T2DM.

All statistical analyses involved were executed in SAS 9.4 or Stata/MP 14.0. Effect sizes were considered to meet statistical significance if two‐sided *p* < .05.

## 3 RESULTS

Baseline demographic characteristics for 241 pairs with an average age of 52.16 ± 9.73, female‐dominated (64.32%), are shown in Table 1. Individuals in the case group showed higher BMI, WC, SBP, DBP, TG, and FPG levels but lower HDL‐C levels than controls. Cases had a higher frequency of obesity, hypertension, and dyslipidemia than controls (all *p* < .05); however, educational level, smoking, alcohol drinking, physical activity, TC level, LDL‐C level, and family history of T2DM did not differ between the two groups.

**TABLE 1:** Baseline characteristics of type 2 diabetes mellitus cases and controls.

Characteristics	Controls (*n* = 241)	Cases (*n* = 241)	*p* value
Age (years)	52.16 ± 9.73	52.16 ± 9.73	—
Female (*n*, %)	155 (64.32)	155 (64.32)	—
High school or above (*n*, %)	21 (8.71)	26 (10.79)	.411^a^
Smoking (*n*, %)	66 (27.39)	58 (24.07)	.194^a^
Alcohol drinking (*n*, %)	24 (9.96)	26 (10.79)	.739^a^
Physical activity (*n*, %)			.583^a^
Moderate/High	175 (72.61)	170 (70.54)	
Low	66 (27.39)	71 (29.46)	
BMI (kg/m^2^)	24.59 (21.98–26.70)	25.67 (23.51–28.50)	<.001^b^
WC (cm)	83.62 ± 10.09	88.49 ± 10.86	<.001^c^
SBP (mm Hg)	124.00 (114.00–138.67)	129.67 (116.67–144.33)	<.001^b^
DBP (mm Hg)	78.33 (71.00–86.33)	81.00 (74.67–92.00)	<.001^b^
TC (mmol/L)	4.47 (3.96–5.01)	4.58 (4.04–5.18)	.110^b^
TG (mmol/L)	1.39 (1.02–1.93)	1.72 (1.15–2.37)	<.001^b^
HDL‐C (mmol/L)	1.16 (0.99–1.34)	1.09 (0.96–1.25)	<.001^b^
LDL‐C (mmol/L)	2.60 (2.20–3.00)	2.60 (2.20–3.10)	.581^b^
FPG (mmol/L)	5.28 (5.05–5.64)	5.95 (5.46–6.42)	<.001^b^
Obesity (*n*, %)	135 (56.02)	171 (70.95)	<.001^a^
Hypertension (*n*, %)	77 (31.95)	109 (45.23)	<.001^a^
Dyslipidemia (*n*, %)	106 (43.98)	131 (54.36)	<.001^a^
Family history of T2DM (*n*, %)	12 (6.15)	21 (11.23)	.095^a^

*Note*: Data are expressed as mean ± SD, median (interquartile range), or *n* (%) as appropriate.

Abbreviations: BMI, body mass index; DBP, diastolic blood pressure; FPG, fasting plasma glucose; HDL‐C, high‐density lipoprotein cholesterol; LDL‐C, low‐density lipoprotein cholesterol; SBP, systolic blood pressure; TC, total cholesterol; TG, triglycerides; WC, waist circumference.

^a^ McNemar's test.

^b^ Wilcoxon signed‐rank test.

^c^ Paired *t* test.

A total of 10 CpG sites were identified by conditional logistic regression. The impact sizes (ORs) of the selected CpG sites on T2DM varied from 1.54 for CpG15 in ABCG1 to 2.98 for CpG3 in TXNIP, details are in Table 2. More details about methylation loci can be found in Table S1. The ranges of simple MRS and weighted MRS were from 0 to 20, and 430.77 to 880.06, respectively. Compared to controls, participants with onset T2DM showed higher simple and weighted MRSs (*p <* .05). Simple MRS was divided into three levels according to the score tertiles: tertile 1 (<7), tertile 2 (7–10), and tertile 3 (≥ 10). Similarly, weighted MRS was classified into tertile 1 (<581.46), tertile 2 (581.46–629.20), and tertile 3 (≥ 629.20).

**TABLE 2:** CpG sites selected to build MRS for type 2 diabetes mellitus.

Gene	Loci	Chromatin position	OR (95% CI)	*p* value
ABCG1				
	CpG6	Chr21: 43656332	1.80 (1.16–2.79)	.008
	CpG15	Chr21: 43656653	1.54 (1.02–2.33)	.038
FTO				
	CpG9	Chr16: 53703794	1.76 (1.16–2.68)	.008
KCNQ1				
	CpG11	Chr11:2445571	1.72 (1.05–2.81)	.030
	CpG41	Chr11:2445330	1.72 (1.05–2.81)	.030
TXNIP				
	CpG1	Chr1: 145441393	2.11 (1.32–3.36)	.002
	CpG2	Chr1: 145441412	1.94 (1.28–2.96)	.002
	CpG3	Chr1: 145441517	2.98 (1.86–4.79)	<.001
	CpG4	Chr1: 145441526	2.03 (1.29–3.18)	.002
	CpG5	Chr1: 145441552	2.03 (1.33–3.10)	.001

Abbreviations: ABCG1, ATP‐binding cassette; CI, confidence interval; FTO, fat mass and obesity associated gene; KCNQ1, potassium voltage‐gated channel member 1 gene; MRS, methylation risk score; OR, odds ratio; TXNIP, thioredoxin‐interacting protein.

Table 3 shows the relationships between the two scores and T2DM onset risk. For simple MRS, after controlling for educational level, smoking, alcohol drinking, physical activity, and FPG, with tertile 1 as reference, the OR and 95% CI of incident T2DM was 2.39 (1.36–4.20) for participants involving tertile 3. For weighted MRS, the risk of T2DM incidence was 2.59 (1.45–4.63) for tertile 3 (model 3). The risk of T2DM showed an increasing trend with higher MRSs (all *P*_for trend_ < .05), whereas with per 1 SD increment in MRS the risk values were 1.66 (1.29–2.14) for simple MRS and 1.60 (1.24–2.08) for weighted MRS.

**TABLE 3:** Association of simple and weighted MRS with type 2 diabetes mellitus.

	Case	Incidence rate, %	OR (95% CI)		
			Model 1	Model 2	Model 3
Simple MRS					
Tertile 1	65	40.63	1.00 (ref)	1.00 (ref)	1.00 (ref)
Tertile 2	49	39.20	0.93 (0.57–1.50)	0.90 (0.55–1.47)	0.86 (0.48–1.54)
Tertile 3	127	64.47	2.69 (1.71–4.23)	2.77 (1.74–4.43)	2.39 (1.36–4.20)
*P*_trend_			<0.001	<0.001	0.003
Per SD increase			1.71 (1.39–2.11)	1.75 (1.41–2.17)	1.66 (1.29–2.14)
Weighted MRS					
Tertile 1	61	38.13	1.00 (ref)	1.00 (ref)	1.00 (ref)
Tertile 2	75	46.30	1.33 (0.84–2.09)	1.29 (0.81–2.04)	1.15 (0.66–1.99)
Tertile 3	105	65.63	3.11 (1.93–5.03)	3.19 (1.95–5.23)	2.59 (1.45–4.63)
*P*_trend_			<0.001	<0.001	0.002
Per SD increase			1.65 (1.34–2.03)	1.68 (1.35–2.09)	1.60 (1.24–2.08)

*Note*: Data are odds ratios (ORs) and 95% confidence intervals (CIs). Model 1: unadjusted. Model 2: adjusted for educational level, smoking, alcohol drinking, and physical activity. Model 3: adjusted for educational level, smoking, alcohol drinking, physical activity, and fasting plasma glucose.

Abbreviation: MRS, methylation risk score.

**FIGURE 1:**
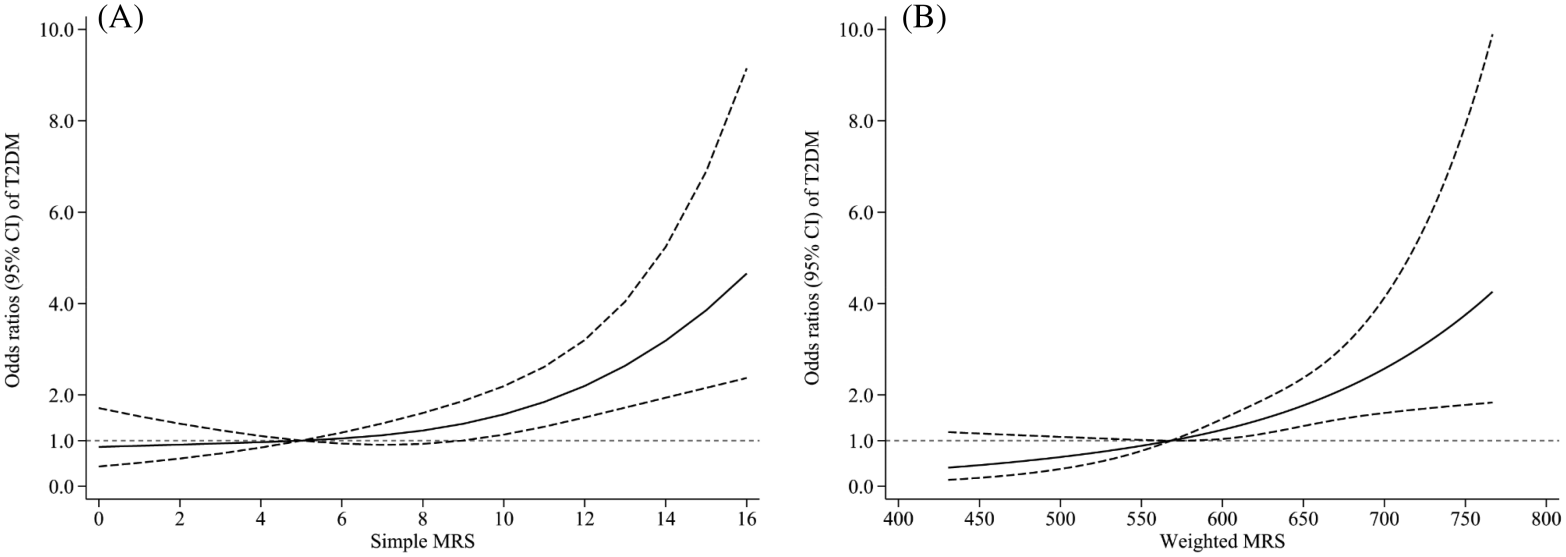
Dose–response associations of simple (A) and weighted (B) methylation risk score (MRS) with risk of type 2 diabetes mellitus. Adjusted for educational level, smoking, alcohol drinking, physical activity, and fasting plasma glucose. CI, confidence interval; T2DM, type 2 diabetes mellitus.

Restricted cubic spline analyses revealed significant J‐curved associations (all *P*_nonlinearity_ < .001) of simple and weighted MRSs with T2DM onset risk in the multivariable‐adjusted model (Figure 1). As MRS elevated, the adjusted T2DM incidence risk increased significantly compared with a simple MRS of 5 and a weighted MRS of 568.36.

As shown in Table 4, we detected significant multiplication interactions among smoking, hypertension, and simple MRS for T2DM onset risk (all *P*_interaction_ < .05). We also found significant multiplication interactions of weighted MRS with smoking and obesity for the risk of T2DM incidence (all *P*_interaction_ < 0.05), with the effect size stronger in participants who were nonsmokers, obese, and diagnosed with hypertension. However, no significant interaction was observed among other confounding factors. Details are shown in Table S2. We calculated the area under the ROC curve (AUC), and the results suggested that the predictive capacity did not differ between simple MRS (AUC = 0.640) and weight MRS (AUC = 0.635). Details are shown in Figure S1.

**TABLE 4:** Stratified analyses of simple and weighted MRS with risk of type 2 diabetes mellitus.

	Simple MRS		*P*_interaction_	Weighted MRS		*P*_interaction_
	Case/N	OR (95% CI)		Case/N	OR (95% CI)	
Smoking			.046			.030
Ever/current						
Tertile 1	17/38	1.00 (ref)		17/37	1.00 (ref)	
Tertile 2	9/25	0.22 (0.03–1.47)		12/34	0.36 (0.07–1.88)	
Tertile 3	32/58	0.68 (0.20–2.340)		29/53	0.68 (0.19–2.44)	
*P*_trend_		.88			.81	
Never						
Tertile 1	48/122	1.00 (ref)		44/123	1.00 (ref)	
Tertile 2	40/97	0.86 (0.40–1.85)		63/128	1.57 (0.78–3.15)	
Tertile 3	95/139	3.76 (1.68–8.40)		76/107	3.95 (1.73–9.02)	
*P*_trend_		.002			.001	
Obesity			0.150			.026
Yes						
Tertile 1	42/103	1.00 (ref)		35/98	1.00 (ref)	
Tertile 2	33/69	0.58 (0.18–1.90)		56/103	1.83 (0.59–5.70)	
Tertile 3	96/134	2.94 (1.04–8.36)		80/105	7.05 (2.10–23.68)	
*P*_trend_		.067			.002	
No						
Tertile 1	23/57	1.00 (ref)		26/62	1.00 (ref)	
Tertile 2	16/56	0.55 (0.11–2.81)		19/59	0.39 (0.08–1.86)	
Tertile 3	31/63	1.92 (0.46–8.00)		25/55	0.87 (0.20–3.77)	
*P*_trend_		.346			.758	
Hypertension			.016			.183
Yes						
Tertile 1	22/56	1.00 (ref)		22/53	1.00 (ref)	
Tertile 2	23/47	1.05 (0.22–4.89)		38/68	2.74 (0.47–16.11)	
Tertile 3	64/83	15.35 (1.70–138.47)		49/65	34.68 (2.40–501.98)	
*P*_trend_		.015			.007	
No						
Tertile 1	43/104	1.00 (ref)		39/107	1.00 (ref)	
Tertile 2	26/78	0.71 (0.31–1.65)		37/94	1.05 (0.47–2.36)	
Tertile 3	63/114	1.26 (0.55–2.89)		56/95	1.84 (0.74–4.54)	
*P*_trend_		0.551			0.211	

*Note*: Adjusted for educational level, smoking, alcohol drinking, physical activity, and fasting plasma glucose except when used as a subgroup variable.

Abbreviations: CI, confidence interval; MRS, methylation risk score; OR, odds ratio.

## 4 DISCUSSION

To our knowledge, this is the first prospective nested case–control study based on the Chinese population to explore the relationship of MRS with T2DM risk. When compared with tertile 1, the risk of incident T2DM in participants involving tertile 3 was 2.39 times higher for simple MRS and 2.59 times greater for weighted MRS. Dose–response curves showed J‐curved associations between simple MRS and weighted MRS and risk of T2DM. In addition, smoking and suffering from hypertension may modify the association between simple MRS and T2DM risk, as may the association between smoking and obesity in weighted MRS and T2DM risk. Our study provides the possibility of identifying population with high genetic risk who would benefit from lifestyle improvement to prevent the development of T2DM.

Recently, despite the use of MRS to predict the risk of individuals developing a chronic disease having increased,^32^, ^33^, ^34^, ^35^ only a handful of studies have investigated the association between MRS and T2DM. In an EWAS of BMI based on 5387 European and Indian Asian individuals, Wahl et al explored the relationship between weighted MRS, which was calculated based on 38 loci, and the risk of T2MD incidence, showing that with per 1 SD increase in MRS the relative risk of T2DM incidence was 2.30 (2.07–2.56).^23^ Additionally, in a nested case–control study, also based on European and Indian Asian participants with an 8‐year follow‐up, which focused on the combining effect for five loci, Chambers et al found that individuals involving quartile 4 of methylation had a 3.51 (2.79–4.42) times higher risk of T2DM incidence than those with quartile 1.^15^ In our previous studies, we found that the hypermethylation of ABCG1,^18^ FTO,^19^ KCNQ1,^20^ and hypomethylation of TXNIP^21^ contributed to a higher risk of T2DM incidence. In our current study, therefore we constructed MRS based on the aforementioned genes. Our study has confirmed the association in the Chinese population: elevated simple and weighted MRSs are associated with increased risk of T2DM.

A few possible mechanisms may explain the positive association of MRS based on ABCG1, FTO, KCNQ1, and TXNIP with the risk of T2DM. DNA methylation of ABCG1 decreases the activity of gene expression resulting in an increase in outflow of cholesterol and an increase in serum levels of fasting insulin and homeostatic model assessment of insulin resistance (HOMA‐IR).^36^, ^37^ In 2007, FTO, identified as the first gene associated with susceptibility to obesity in a genome‐wide association study, could well lead to diabetes mellitus through the effect on BMI.^38^, ^39^ A child‐based case–control study found that FTO methylation was positively associated with WC and body fat, which greatly contribute to the incidence of T2DM.^40^ Compared to non‐T2DM participants, those who had T2DM^41^ experienced different KCNQ1 methylation in their fatty tissue. Hypermethylation of KCNQ1 decreased insulin sensitivity.^42^ Hypomethylation of TXNIP was negatively associated with FPG, HOMA‐IR levels and an increased risk of T2DM.^43^

Epigenetic provides a link between environmental factors and gene activity.^10^, ^14^ Smoking substantially alters methylation levels at many CpG loci in the genome, increasing the risk of T2DM.^44^, ^45^ In simple and weighted MRSs, however, we found a stronger association between tertile 3 participants and the risk of incident T2DM in nonsmokers compared to the ever/current smoking group, which could be explained by endothelial dysfunction^46^ and increase in inflammation^47^ caused by smoking, possibly masking the relationship between MRS and T2DM. Obesity is an important factor driving T2DM, for which BMI is a key measure. An epigenome‐wide association study conducted by Wahl et al identified 187 CpG sites whose methylation levels were affected by BMI, demonstrating that obesity is the cause of altered DNA methylation.^23^ This is in line with our results, which showed that in weighted MRS there was a stronger association between participants in tertile 3 and the risk of T2DM incidence in obese participants, taking normal weight participants as reference. The multiplication interaction between simple MRS and hypertension for risk of T2DM incidence could be explained as impaired glucose tolerance, which is closely associated with hypertension,^48^ while Janghorbani et al suggested that high plasma glucose levels were independently associated with hypertension incidence.^49^ It is of interest that an exercise intervention study conducted by Nitert et al found that after 6 months of exercise the methylation levels of 134 individual genes changed, 115 of which showed decreased methylation regardless of family history of T2DM.^50^ In the current study, however, we found no modification effect of physical activity on the association between MRS and T2DM, possibly due to the small sample size. The reversible nature of epigenetic modifications indicates that they are a promising target for future T2DM treatment to which we should pay more attention.^10^ People who smoke, are obese, have hypertension, and have low physical activity levels should improve their lifestyle in time to actively prevent T2DM.

In our current study, we constructed MRS based on the methylation of ABCG1, FTO, KCNQ1, and TXNIP before T2DM occurred, which reduced recall bias. Participants were identified from the same cohort and matched, which reduced selection bias. Further, we used MRS to explore the relationship of the collective effect of genes methylation level and T2DM incidence risk. The significant dose–response relationships and interactions helped us better understand the association.

Some limitations could have affected our findings. First, we only extracted DNA from white blood cells in whole blood and did not take the variation in methylation levels between different tissues into account. Second, we calculated MRS based on methylation levels at baseline, whereas individual DNA methylation changes occurred in response to lifestyle and environmental factors. Further research is needed here. Finally, the individuals involved in the research were sampled from the middle of China, which limits the representativeness of our findings. Future studies based on multiethnic samples are therefore needed to validate the current findings.

## 5 CONCLUSION

In conclusion, we found that elevated simple and weighted MRSs were associated with an increase in T2DM risk and that environmental risk factors may influence the association between MRS and T2DM.

## DISCLOSURE

No conflict.

## Supporting information


**Figure S1.** Receiver operator characteristic curves for simple methylation risk score (MRS) and weighted MRS in predicting type 2 diabetes mellitus (T2DM).
**Table S1.** Association of methylation level of loci with type 2 diabetes mellitus.
**Table S2.** Stratified analyses of methylation risk score (MRS) with risk of type 2 diabetes mellitus.
