# Supplementary material for: Association of methylation risk score with incident type 2 diabetes mellitus: A nested case–control study
Source: J Diabetes. 2023 Dec 7;16(3):e13512. doi: 10.1111/1753-0407.13512 (PMC10940902; doi:10.1111/1753-0407.13512)
Supplement: Supplementary file 1 — Figure S1. Receiver operator characteristic curves for simple methylation risk score (MRS) and weighted MRS in predicting type 2 diabetes mellitus (T2DM). Table S1. Association of methylation level of loci with type 2 diabetes mellitus. Table S2. Stratified analyses of methylation risk score (MRS) with risk of type 2 diabetes mellitus. [file JDB-16-e13512-s001.docx]

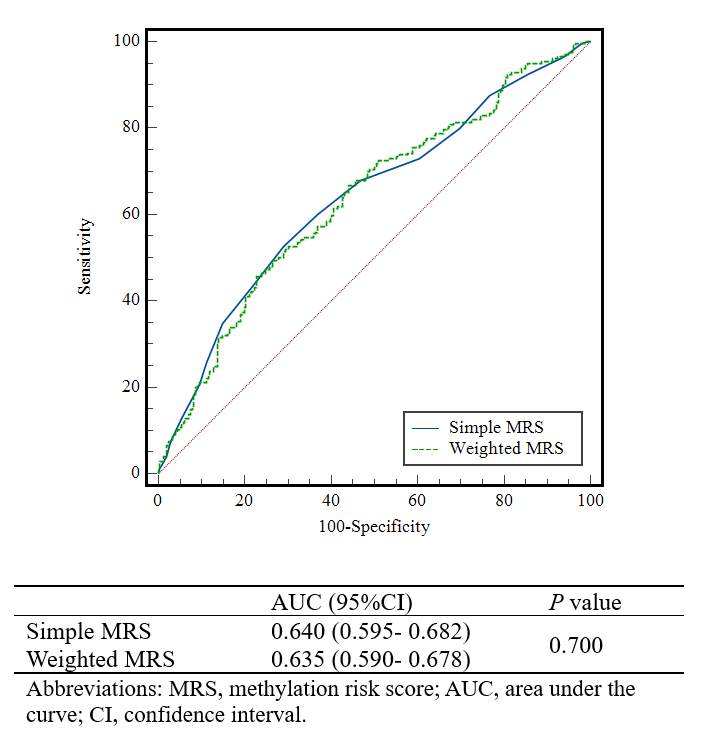


**Supplementary Figure 1.** Receiver Operator Characteristic Curves for simple MRS and weighted MRS in predicting T2DM.

| **Supplementary Table 1.** Association of methylation level of loci with type 2 diabetes mellitus | | | | |
| --- | --- | --- | --- | --- |
| Gene | Loci | Chromatin Position | OR (95% CI) | *P* value |
| ABCG1 |  |  |  |  |
|  | CpG1 | Chr21: 43656282 | 0.82 (0.53-1.27) | 0.373 |
|  | CpG2 | Chr21: 43656291 | 0.82 (0.53-1.27) | 0.373 |
|  | CpG3 | Chr21: 43656299 | 1.13 (0.72-1.77) | 0.592 |
|  | CpG4 | Chr21: 43656310 | 0.79 (0.52-1.21) | 0.283 |
|  | CpG11 | Chr21: 43656535 | 0.88 (0.61-1.25) | 0.469 |
|  | CpG12 | Chr21: 43656546 | 1.22 (0.82-1.84) | 0.326 |
|  | CpG13 | Chr21: 43656587 | 1.43 (0.92-2.21) | 0.111 |
|  | CpG14 | Chr21: 43656590 | 1.43 (0.92-2.21) | 0.111 |
| FTO |  |  |  |  |
|  | CpG1 | Chr16:53703596 | 1.31 (0.84-2.05) | 0.238 |
|  | CpG2 | Chr16:53703611 | 1.11 (0.69-1.77) | 0.678 |
|  | CpG3 | Chr16:53703666 | 1.13 (0.74-1.74) | 0.565 |
|  | CpG4 | Chr16:53703708 | 0.80 (0.56-1.15) | 0.232 |
|  | CpG5 | Chr16:53703716 | 0.91 (0.62-1.35) | 0.655 |
|  | CpG6 | Chr16:53703731 | 1.08 (0.69- 1.69) | 0.729 |
|  | CpG7 | Chr16: 53703736 | 1.08 (0.69- 1.69) | 0.729 |
|  | CpG8 | Chr16: 53703738 | 1.08 (0.69- 1.69) | 0.729 |
|  | CpG10 | Chr16:53703805 | 1.20 (0.78-1.85) | 0.415 |
|  | CpG11 | Chr16:53703832 | 0.95 (0.59-1.51) | 0.812 |
|  | CpG12 | Chr16:53703843 | 1.00 (0.66-1.51) | 0.997 |
|  | CpG13 | Chr16: 53703846 | 1.00 (0.66-1.51) | 0.997 |
|  | CpG14 | Chr16:53703852 | 0.76 (0.47-1.24) | 0.276 |
|  | CpG15 | Chr16: 53703857 | 0.76 (0.47-1.24) | 0.276 |
|  | CpG16 | Chr16: 53703861 | 0.76 (0.47-1.24) | 0.276 |
|  | CpG17 | Chr16: 53703869 | 0.76 (0.47-1.24) | 0.276 |
|  | CpG18 | Chr16:53703880 | 0.92 (0.62-1.35) | 0.655 |
|  | CpG19 | Chr16:53703902 | 1.03 (0.68-1.55) | 0.907 |
| KCNQ1 |  |  |  |  |
|  | CpG1 | chr11:2445715 | 0.84 (0.53-1.33) | 0.455 |
|  | CpG2 | chr11:2445701 | 1.11 (0.76-1.62) | 0.575 |
|  | CpG3 | chr11: 2445698 | 1.11 (0.76-1.62) | 0.575 |
|  | CpG4 | chr11: 2445690 | 1.11 (0.76-1.62) | 0.575 |
|  | CpG5 | chr11:2445680 | 0.98 (0.63-1.54) | 0.934 |
|  | CpG6 | chr11:2445660 | 0.92 (0.56-1.52) | 0.754 |
|  | CpG10 | chr11:2445574 | 1.06 (0.69-1.62) | 0.802 |
|  | CpG12 | chr11:2445559 | 0.96 (0.61-1.50) | 0.855 |
|  | CpG19 | chr11:2445483 | 0.76 (0.48-1.20) | 0.245 |
|  | CpG20 | chr11:2445480 | 0.76 (0.48-1.20) | 0.245 |
|  | CpG22 | chr11:2445453 | 1.28 (0.83-1.97) | 0.270 |
|  | CpG23 | chr11:2445445 | 0.94 (0.61-1.44) | 0.767 |
|  | CpG24 | chr11:2445441 | 0.94 (0.61-1.44) | 0.767 |
|  | CpG25 | chr11:2445426 | 0.94 (0.63-1.40) | 0.758 |
|  | CpG26 | chr11:2445424 | 0.94 (0.63-1.40) | 0.758 |
|  | CpG27 | chr11:2445414 | 0.92 (0.60-1.41) | 0.702 |
|  | CpG28 | chr11: 2445406 | 0.92 (0.60-1.41) | 0.702 |
|  | CpG29 | chr11: 2445404 | 0.92 (0.60-1.41) | 0.702 |
|  | CpG30 | chr11: 2445395 | 1.17 (0.74-1.87) | 0.505 |
|  | CpG31 | chr11: 2445391 | 1.17 (0.74-1.87) | 0.505 |
|  | CpG32 | chr11: 2445388 | 1.17 (0.74-1.87) | 0.505 |
|  | CpG33 | chr11: 2445386 | 1.17 (0.74-1.87) | 0.505 |
|  | CpG34 | chr11: 2445369 | 0.98 (0.63-1.53) | 0.927 |
|  | CpG35 | chr11: 2445367 | 0.98 (0.63-1.53) | 0.927 |
|  | CpG36 | chr11: 2445365 | 0.98 (0.63-1.53) | 0.927 |
|  | CpG37 | chr11: 2445350 | 1.13 (0.73-1.76) | 0.574 |
|  | CpG38 | chr11: 2445347 | 1.13 (0.73-1.76) | 0.574 |
|  | CpG39 | chr11: 2445343 | 1.13 (0.73-1.76) | 0.574 |
|  | CpG40 | chr11: 2445341 | 1.13 (0.73-1.76) | 0.574 |
|  | CpG49 | Chr11:2445284 | 1.50 (0.96-2.36) | 0.075 |
|  | CpG50 | Chr11: 2445282 | 1.50 (0.96-2.36) | 0.075 |
|  | CpG51 | Chr11: 2445272 | 1.29 (0.81-2.05) | 0.282 |
|  | CpG52 | Chr11: 2445269 | 1.29 (0.81-2.05) | 0.282 |
|  | CpG53 | Chr11: 2445265 | 1.29 (0.81-2.05) | 0.282 |
|  | CpG54 | Chr11: 2445263 | 1.29 (0.81-2.05) | 0.282 |
|  | CpG55 | Chr11:2445256 | 1.25 (0.77-2.03) | 0.369 |
|  | CpG56 | Chr11: 2445254 | 1.25 (0.77-2.03) | 0.369 |
| Abbreviations: MRS, methylation risk score; OR, odds ratio; CI, confidence interval. | | | | |

| **Supplementary Table 2.** Stratified analyses of MRS with risk of type 2 diabetes mellitus | | | | | | |
| --- | --- | --- | --- | --- | --- | --- |
|  | Simple Methylation Risk Score | | *P*_interaction_ | Weighted Methylation Risk Score | | *P*_interaction_ |
|  | Case/N | OR (95% CI) |  | Case/N | OR (95% CI) |  |
| **Gender** |  |  | 0.066 |  |  | 0.068 |
| Female |  |  |  |  |  |  |
| Tertile 1 | 44/114 | 1.00 (ref) |  | 39/113 | 1.00 (ref) |  |
| Tertile 2 | 32/81 | 0.81 (0.37-1.79) |  | 56/111 | 1.59 (0.78-3.26) |  |
| Tertile 3 | 79/115 | 3.99 (1.73-9.20) |  | 60/86 | 4.21 (1.78-9.93) |  |
| *P*_trend_ |  | 0.002 |  |  | 0.001 |  |
| Male |  |  |  |  |  |  |
| Tertile 1 | 21/46 | 1.00 (ref) |  | 22/47 | 1.00 (ref) |  |
| Tertile 2 | 17/44 | 0.84 (0.31-2.26) |  | 19/51 | 0.53 (0.19-1.49) |  |
| Tertile 3 | 48/82 | 1.25 (0.53-2.95) |  | 45/74 | 1.28 (0.54-3.03) |  |
| *P*_trend_ |  | 0.567 |  |  | 0.474 |  |
| **Physical**  **Activity** |  |  | 0.899 |  |  | 0.906 |
| Moderate/high |  |  |  |  |  |  |
| Tertile 1 | 47/122 | 1.00 (ref) |  | 46/121 | 1.00 (ref) |  |
| Tertile 2 | 35/86 | 1.00 (0.39-2.53) |  | 48/110 | 1.24 (0.55-2.81) |  |
| Tertile 3 | 88/137 | 2.54 (1.16-5.58) |  | 76/114 | 3.40 (1.47-7.89) |  |
| *P*_trend_ |  | 0.014 |  |  | 0.005 |  |
| Low |  |  |  |  |  |  |
| Tertile 1 | 18/38 | 1.00 (ref) |  | 15/39 | 1.00 (ref) |  |
| Tertile 2 | 14/39 | 0.616 (0.12-3.12) |  | 27/52 | 1.5 (0.33-7.26) |  |
| Tertile 3 | 39/60 | 5.34 (0.70-40.51) |  | 29/46 | 4.15 (0.50-34.64) |  |
| *P*_trend_ |  | 0.135 |  |  | 0.161 |  |
| **Dyslipidemia** |  |  | 0.259 |  |  | 0.495 |
| Yes |  |  |  |  |  |  |
| Tertile 1 | 35/71 | 1.00 (ref) |  | 31/71 | 1.00 (ref) |  |
| Tertile 2 | 23/62 | 0.75 (0.16-3.63) |  | 37/76 | 0.81 (0.08-7.74) |  |
| Tertile 3 | 73/104 | 3.72 (0.86-16.02) |  | 63/90 | 9.36 (1.64-53.42) |  |
| *P*_trend_ |  | 0.062 |  |  | 0.009 |  |
| No |  |  |  |  |  |  |
| Tertile 1 | 30/89 | 1.00 (ref) |  | 30/89 | 1.00 (ref) |  |
| Tertile 2 | 23/63 | 0.87 (0.22-3.38) |  | 38/86 | 0.90 (0.26-3.11) |  |
| Tertile 3 | 54/93 | 1.59 (0.52- 4.81) |  | 42/70 | 1.07 (0.34-3.38) |  |
| *P*_trend_ |  | 0.378 |  |  | 0.928 |  |
| Abbreviations: OR, odds ratio; CI, confidence interval.  Adjusted for educational level, smoking, alcohol drinking, physical activity, and fasting plasma glucose except when used as a subgroup variable. | | | | | | |
